# Self-reported recognition of undiagnosed life threatening conditions in chiropractic practice: a random survey

**DOI:** 10.1186/2045-709X-20-21

**Published:** 2012-07-05

**Authors:** Dwain M Daniel, Harrison Ndetan, Ronald L Rupert, Daniel Martinez

**Affiliations:** 1 Texas Chiropractic College, Pasadena, TX, USA; 2 Parker University, Dallas, TX, USA

**Keywords:** Chiropractic, Education, Professional role, Primary care, Diagnosis, Life threatening

## Abstract

**Background:**

The purpose of this study was to identify the type and frequency of previously undiagnosed life threatening conditions (LTC), based on self-reports of chiropractic physicians, which were first recognized by the chiropractic physician. Additionally this information may have a preliminary role in determining whether chiropractic education provides the knowledge necessary to recognize these events.

**Methods:**

The study design was a postal, cross-sectional, epidemiological self-administered survey. Two thousand Doctors of Chiropractic in the US were randomly selected from a list of 57878. The survey asked respondents to state the number of cases from the list where they were the first physician to recognize the condition over the course of their practice careers. Space was provided for unlisted conditions.

**Results:**

The response rate was 29.9%. Respondents represented 11442 years in practice and included 3861 patients with a reported undiagnosed LTC. The most commonly presenting conditions were in rank order: carcinoma, abdominal aneurysm, deep vein thrombosis, stroke, myocardial infarction, subdural hematoma and a large group of other diagnoses. The occurrence of a previously undiagnosed LTC can be expected to present to the chiropractic physician every 2.5 years based on the responding doctors reports.

**Conclusion:**

Based on this survey chiropractic physicians report encountering undiagnosed LTC’s in the normal course of practice. The findings of this study are of importance to the chiropractic profession and chiropractic education. Increased awareness and emphasis on recognition of LTC is a critical part of the education process and practice life.

## Background

There are several different definitions of what constitutes a primary care physician [1,2]. Most of these definitions incorporate the concept of “assumption of longitudinal responsibility for the patient regardless of the presence or absence of disease” [3]. Based on this definition a physician, at a minimum, has the moral and legal responsibility to be able to identify and refer, when necessary, conditions which if left unrecognized and untreated may result in serious and even deadly consequences to the patient.

There is an ongoing controversy within the healthcare community regarding role of the chiropractic physician as well as debates on this issue within the chiropractic profession itself. There are three basic positions argued; primary care, neuromusculoskeletal care and subluxation based care. The Council on Chiropractic Education mandates that chiropractic educational institutions “train a competent doctor of chiropractic who will provide quality patient care and serve as a primary care physician” [4]. Duenas et al. found 94% of chiropractic colleges utilize the term “primary care provider” or similar variants when describing the product of their educational programs [1]. In spite of primary care training some argue the proper role of the chiropractor is as a neuromusculoskeletal or musculoskeletal specialist [5,6]. This position is supported at least in part by the profile of the typical chiropractic patient. According to The National Board of Chiropractic Examiners, 82.9% of presenting complaints in the typical chiropractor’s office consist of back pain, extremity pain, neck pain and headache. Less than 10% of patients present with non-neuromusculoskeletal complaints [7]. A third position argues chiropractic is a separate and distinct health care specialty that should limit itself to detecting and correcting subluxations [8].

The training students receive in chiropractic college is broad based and similar to medical training [9]. A 2005 study tested 4th year chiropractic students and 4^th^ year medical students on their knowledge of primary care activities. Overall chiropractic students scored slightly lower than medical students in all areas but musculoskeletal diagnosis [10]. Considering the differences on emphasis relating to primary care between the disciplines the outcomes are not surprising, except possibly that the chiropractic student did so well compared to their medical counterpart. Additionally, a 1995 survey of 753 chiropractors reported 78.5% of respondents considered their training as primary care providers as “adequate” [11].

It has been suggested chiropractic training is deficient to medical training for primary care due to the lack of patient contact [12]. The chiropractic student usually encounters patients with musculoskeletal conditions or asymptomatic patients presenting for wellness care during their internship [13]. Although a medical doctor can be granted a license to practice with as little as 1 year in a post graduate residency [14], most have a minimum of 2 to 3 years in a residency program. This additional exposure provides patient contact with a wide variety of conditions. Essentially the chiropractic physician is limited to knowledge gained in the classroom and limited patient contact. Whether this difference produces a physician that is insufficiently trained to recognize undiagnosed life threatening conditions (LTC) has not been determined.

A review of the Job *Analysis of Chiropractic* reveals that chiropractors have contact with patients with a wide variety of serious conditions and often co-manage these patients with a medical provider [7]. What is not included in the analysis is whether the chiropractor was the first to recognize these conditions in an undiagnosed patient.

There is relatively little information in the scientific literature relating to the chiropractic physician’s ability to recognize LTC. The purpose of this paper is to begin the process to determine whether chiropractic training equips the chiropractic physician to recognize conditions which were previously undiagnosed, may be fatal if left untreated and usually fall outside the scope of chiropractic practice. Thus, the specific purpose was to identify the type and frequency of self-reported cases of undiagnosed life threatening conditions which doctors of chiropractic claim have presented in their offices during their careers. If these conditions were commonly reported, it was also of interest to explore any potential associations between college of graduation, years in practice, type of practice or prior medical training and the rate of recognition.

## Methods

The study was a self-administered, epidemiological survey involving randomly selected doctors of chiropractic (DC) practicing in the United States. The survey instrument was a self-administered one paged questionnaire that asked DCs to state the number of cases (patients), based on their recall over the course of their careers, which presented in their offices with LTC which had not been previously diagnosed or recognized. Included in the survey was a list of the following conditions: carcinoma, abdominal aneurysm, deep vein thrombosis, stroke, myocardial infarction, subdural hematoma and other. This list was developed from an earlier unpublished study of chiropractors which did not include a list of conditions. The operational definition of “life threatening” provided to the doctor was “an illness or injury that, if it remains unrecognized and untreated, could result in the death on the individual within 24 months.” The initial and follow up survey included a request to complete the survey even if the doctor had never encountered a LTC in their practice. Based on the responses given it is possible some doctors did not read or ignored the definition of “life threatening condition” the authors provided. There was evidence this occurred in some responses. In reviewing the surveys the authors attempted to correct for these errors. The authors recognize their exclusions were not systematic. Since this is a survey and by nature biased, the authors did not feel it necessary to develop a system to rule out specific responses. The authors used their training as physicians to eliminate those responses that were, in their opinion, not life threatening or excessive reporting. Data were reviewed prior to statistical analysis and data points that were determined to be invalid were excluded.

The survey also solicited demographic information such as gender, college of graduation of the doctor and whether or not they have had any prior health care experience. They were also asked to provide information on some practice characteristics such as number of years in practice, type of practice (solely subluxation-based or not), and practice settings (rural, suburban, urban, solo, multi-DC, or multi-discipline practice) as well as whether or not they were employed by the Veteran Administration (VA) or the military. In early November 2010, 2000 doctors were randomly selected by computer program from a mailing list of 57878 chiropractors provided by MPA Media. Two mailings to each doctor were performed over a two month period. The study was approved by both the research committee and the institutional review board of the institution where this research was conducted.

### Statistical method

All survey responses were entered into a PASW statistics 18 spread sheet (SPSS Inc., Chicago, IL) for analysis. For the purpose of analysis, a response variable ‘rate of occurrence’ (the number of undiagnosed LTC (count) per year in practice) was defined as the ratio of the number of cases identified to the number of years in practice for each respondent. This variable was defined for each of the 6 specific conditions listed as being most common on the current survey (carcinoma, myocardial infarction, stroke, subdural hematoma, abdominal aneurism, and deep vein thrombosis), all other cases seen combined, and for the total number of cases seen in general. The colleges of graduation were grouped into three categories based on the cultures of the colleges as ‘liberal, mixed or conservative’ based on information provided in a report developed by the Institute for Alternative Futures [15]. Years of experience was also categorized into three levels: “education dominant” (less than 5 years in practice), “education and experience equal” (above 5 to 15 years in practice), and “experience dominant” (above 15 years in practice). Poisson regression models were used in generating mean ‘rate of occurrence’ for each of the conditions and the total occurrences. In order to identify factors that would predict ‘rate of occurrence’ a Poisson regression model was constructed with ‘rate of occurrence’ for the total number of cases seen in general as the response and all the demographic and practice characteristics as predictors. Statistical significance was assessed at the 5% level. The actual effect estimates generated by the Poisson regression models were mean rates of occurrence (the number of undiagnosed LTC per years in practice) but for the sake of easy comprehension we have reported the reciprocals of these estimates which give the number of years in practice per presentation of an undiagnosed LTC (years-per-case).

## Results

A total of 588 completed surveys were received (a 29.9% response rate) and 35 uncompleted surveys were returned as undeliverable (31), doctor was deceased (2), or retired (2). Of the completed surveys 12 (2%) were eliminatedfrom the analysis due invalid responses. Thirty seven responses were removed from the list of reported LTC which in the opinion of the authors did not qualify as life threatening. Additionally the responses of 12 doctors who reported between 171 to 500 encounters were also excluded as being excessive.

Most of the respondents were male DCs (79.4%) and had been in practice for over 15 years (60.7%). The mean number of years in practice reported was 20.0 +/− 10.5. Most of the DCs reported practicing in a suburban setting (46.3%) and in solo practice (52.3%). No doctor reported working with the Veteran Administration or military. Graduates from Palmer Chiropractic College in Davenport represented the highest percentage of respondents (23.2%). Based on the culture of the colleges of graduation, 61.6% were mixed, 23.7% liberal and 14.8% conservative. The complete list of demographic and practice characteristics of the respondents are shown in Table 1.

**Table 1:** Demographics and practice characteristics for DC’s who responded to the survey on identifying life threatening conditions in their offices [N = 576 (98%)]

	**N(%)**
Gender	
Male	452 (79.4)
Female	117 (20.6)
Years In practice	
Education dominant	27(4.7)
Education/Experience mixed	198 (34.6)
Experience dominant	348 (60.7)
Traditional Chiro	221 (39.7)
Rural setting	128 (22.3)
Suburban	266 (46.3)
Urban	142 (24.7)
Multi-Discipline	59 (10.3)
Veterans	0 (0)
Solo	300 (52.3)
Multi DC	121 (21.0)
Other Healthcare Experience	63 (11.0)
College of Graduation	
Palmer CC Davenport (M)	126 (23.2)
National University (L)	53 (9.8)
Life University (C)	51 (9.4)
Logan (M)	47 (8.7)
New York CC (M)	39 (7.2)
Northwestern HS Univ. (M)	33 (6.1)
Parker CC (M)	32 (5.9)
Southern California Univ. of HS (L)	31 (5.8)
Cleveland CC KS (M)	26 (4.8)
University of Western States CC (L)	21 (3.9)
Texas CC (L)	20 (3.7)
Palmer CC WEST (M)	18 (3.3)
Sherman CC (C)	18 (3.3)
Cleveland LA (M)	11 (2.0)
Life CC WEST (C)	11 (2.0)
University of Bridgeport (L)	3 (.6)
Canadian Memorial CC	1 (.2)
Palmer CC Florida (M)	1 (.2)
Type of College of Graduation (grouped)	
Liberal	128 (23.7)
Mixed	333 (61.6)
Conservative	80 (14.8)

* Top 3 states with at least 30 respondents (DCs) were California (12.1%), New York (5.9%), and Texas (5.4%).

(M) Mixed educational culture, (C) Conservative educational culture, (L) Liberal educational culture.

A total of 470 (81.6%) respondents reported having been the first to identify at least one LTC during their practice career. The maximum number of cases for all conditions in general identified by a single doctor was 71 and a total of 3861 cases were noted by all the respondents combined. Based on the total number of years in practice (11442 years) reported by these respondents, this amounted to an average of 2.5 years-per-case. The conditions reported most (percentage of doctors who reported) were carcinoma (58.9%), abdominal aneurism (43.4%), deep vein thrombosis (29.9%), stroke (27.4%), and myocardial infarction (25.0%). Other conditions commonly reported (21.7%) included severe hypertension, diabetes, meningitis, cervical fracture and appendicitis. Details on the maximum number of cases reported by a single doctor, total number of reported cases by all the doctors, and the mean number of years in practice per case for each of these conditions as well as for other reported conditions are outlined in Table 2. Factors that may be predictive of occurrence, based on self-reports, of a condition presenting in their office include gender of doctor, years in practice, and practice characteristics such as whether or not a doctor practiced traditional chiropractic, practiced in a rural setting or group (multi-DC) setting (Table3).

**Table 2:** Number of doctors reporting conditions, maximum number of cases per doctor, total number of cases by all reporting doctors and the ratio of total year in practice to total cases of life threatening conditions identified in chiropractic offices (N = 576)

**Conditions**	**N (%)**	**Max # of cases**	**Total # of cases**	**Mean (S.D.)**	**Year to Case Ratio**^**%**^	**Year to case ratio**^**#**^
Total (in all)	470 (81.6)	71	3861	6.7 (10.3)	3.0	2.5
Carcinoma	339 (58.9)	38	1226	2.1 (3.6)	9.3	7.8
Abdominal aneurism	250(43.4)	30	856	1.5 (3.3)	13.4	11.8
Deep vein thrombosis	172 (29.9)	40	628	1.1 (3.1)	18.2	15.0
Stroke	158 (27.4)	12	340	0.6 (1.4)	33.7	33.3
Myocardial infarction	144 (25.0)	21	375	0.7 (1.8)	30.5	27.0
Subdural hematoma	48 (8.3)	6	84	0.2 (0.6)	136.2	125.0
Others	125 (21.7)	30	352	0.6 (2.3)	32.5	27.8

*The maximum years in practice reported by all doctors combined was 60, minimum 1, mean 20.0+/−10.5, and total 11442.

% The ratio of sum of years in practice to sum of number of cases.

# Estimation from Poisson Regression analysis.

**Table 3:** Factors that were predictive to number of cases of life threatening conditions a doctor is likely to identify for a given year in practice

**Factors**	**Year-per-case**	**p-value**
Gender		.047*
Male	2.9	
Female	3.4	
Years in Practice		
Education dominant	2.2	.718
Equal education/experience	1.9	<.0001
Experience dominant	3.4	Ref
Traditional chiropractic		.278
Yes	3.7	
No	2.6	
Rural setting		.007*
Yes	2.2	
No	3.3	
Suburban		.731
Yes	2.9	
No	3.0	
Multi-discipline		.462
Yes	3.2	
No	2.9	
Solo		.740
Yes	3.4	
No	2.6	
Multi-DC		.019*
Yes	2.0	
No	3.4	
Other healthcare experience		.668
Yes	2.4	
No	3.1	
Type of college of graduation		
Liberal	2.6	.112
Mixed	2.9	.181
Conservative	3.0	Ref

*Four factors were predictive of rate of occurrence of undiagnosed life threatening conditions in chiropractic practice: years in practice, gender, practicing in a group (multi-DC) and practicing in a rural setting.

## Discussions

The purpose of this study was not to establish whether chiropractic training prepares the chiropractor to be a primary care physician, neuromusculoskeletal specialist or subluxation based provider. The role of the chiropractor in healthcare has been eloquently debated in several articles [1,2,5,16-18]. Our interest was to provide preliminary data in the process of determining if chiropractic training provides the chiropractor the ability to recognize undiagnosed LTC which is a fundamental requirement of all physicians. The 576 surveys validated for our study represent 11442 years in practice and a reported 3861 patients with an undiagnosed LTC. Based on this data the typical chiropractor could expect to have 1 patient with an undiagnosed LTC to present in their office every 2.5 practice years using Poisson Regression analysis. Respondents identified over 50 different conditions that fit the operational definition of “life threatening”. Considering the total number and wide variety of diagnoses reported the authors feel there is preliminary evidence to support the position that chiropractic training provides an educational base to the chiropractor to be able to recognize undiagnosed LTC when they present in their office.

The concern relating to missed or delayed diagnosis is common throughout the healthcare professions. Between 2007 and 2009 the NCMIC Insurance Company reported 7.5% of claims made against their insured chiropractors were related to missed diagnosis or delayed referral based on personal communications with Keith P. Henaman, Assistant Vice President of Claims. Within the medical community the most common malpractice lawsuits were related to missed diagnosis, failure to diagnose or delayed diagnosis [19] representing 34% of primary care cases according to Phillips et al. [20]. Interestingly the most common missed diagnoses in medical practice are similar to the most common presentations of LTC in chiropractor’s offices. These include carcinoma, myocardial infarct, stroke and abdominal aneurysm [20-22]. This is not to suggest chiropractors are diagnosing conditions missed in the medical doctor’s office but that these type conditions are common, easy to misdiagnose and can have deadly consequences. As one author noted the gold standard of diagnosis, the clinical autopsy, would not have been necessary in 12% of cases if the proper diagnosis had been made and the proper treatment given [23].

A very important component of our study was to identify the most common conditions chiropractors reported encountering. In rank order they include carcinoma, abdominal aneurysm, deep vein thrombosis, stroke, myocardial infarction and subdural hematoma. There was a large group of other conditions commonly reported which included severe hypertension, other cardiovascular disease, infection, diabetes, meningitis, cervical fracture and appendicitis. It is interesting to note that many of the most commonly reported presenting conditions often share one of two characteristics. The first is that many patients present with conditions that may manifest themselves as neuromusculoskeletal in origin thereby causing them to choose a chiropractor as the physician of choice. These conditions could include myocardial infarction manifesting as thoracic tightness, carcinoma resulting in perceived muscle/bone pain, deep vein thrombosis resulting in leg pain and stroke or subdural hematoma resulting in headache or extremity weakness. Secondly many of the conditions reported could appear to be discovered incidental to radiographic examination such as abdominal aneurysm and carcinoma.

In addition to the number of LTC reported and the varied diagnoses, several findings of interest were discovered when analyzing relationships (Table 3). The reader is cautioned that the reasons for these differences may be due to the inherent bias of the survey instrument and should be interpreted as no more than points of interest requiring additional study.

1. Although the chiropractic profession continues to battle over philosophical questions, this study provided preliminary evidence that the reported ability to recognize LTC was equal among schools when divided into liberal, mixed and conservative categories. Additionally there was no significant differences in those doctors that categorized themselves as “subluxation based” compared to those that did not.

2. Rural practices appeared to experience an increase in reported recognition of patients with LTC compared to other practice environments.

3. A doctor in multi-DC practice reported recognizing more LTC than a doctor in solo practice.

4. Doctors with a blend of recent education and experience (6 to 15 year’s experience) reported recognizing LTC at a greater rate than more recent graduates or those in practice over 15 years.

5. Male DC’s reported recognizing LTC at a more frequent rate than female DC’s.

This paper had several limitations which are common to survey instruments. Recall bias may have resulted in over reporting or under reporting of LTC.

The presence of LTC was determined by the survey takers themselves and did not require confirmation by any outside source. It is possible that what a responder thought was a LTC may have been misdiagnosed or not truly life threatening and as a result weakened our findings. It should be noted a total of 37 conditions were removed from data analysis because in the opinion of the authors they did not meet the operational definition of life threatening. Two examples of conditions removed included subluxation and mononucleosis. The response rate for this survey was lower than desired. The findings in a study by Russell et al. indicated that the response rates for postal surveys of chiropractors range from 7.0% to 91.4% with an a mean of 52.7% [24]. It should be noted many surveys reported in the Russell et al. study were of specific populations of chiropractors which generally provide higher response rates, while surveys which include all chiropractors generally have lower response rates [25-27].

Lastly this survey did not attempt to determine “missed” diagnoses on the part of the chiropractor as that was not the purpose of the paper. The authors acknowledge that diagnoses may have been missed which would have increased the frequency of these conditions.

## Conclusions

The data from this study provides preliminary evidence that chiropractic education prepares the DC to recognize LTC when they present in their offices. Additionally this study provides preliminary identification and frequency of LTC which commonly present in the chiropractor’s office. This information is important to chiropractic colleges, continuing education programs and the practicing DC. Heightened awareness and knowledge of these conditions should improve patient care by reducing the risk of missed or delayed diagnosis.

As stated previously this is a preliminary study. Additional prospective studies are required to develop more reliable and accurate information in this area of investigation.

## Competing interests

The authors declare that they have no competing interests.

## Authors’ contributions

DD was primarily responsible for design, interpretation of data and drafting of the manuscript. HN was primarily responsible for statistical analysis. RR and DM provided substantial contribution to the design of the study, interpretation of data, revisions and editing of the manuscript. All authors read and approved the final manuscript.
